# Acknowledgement to Reviewers of *Geriatrics* in 2018

**DOI:** 10.3390/geriatrics4010010

**Published:** 2019-01-09

**Authors:** Geriatrics Editorial Office

**Affiliations:** MDPI, St. Alban-Anlage 66, 4052 Basel, Switzerland

Rigorous peer-review is the corner-stone of high-quality academic publishing. The editorial team greatly appreciates the reviewers who contributed their knowledge and expertise to the journal’s editorial process over the past 12 months. In 2018, a total of 91 papers were published in the journal, with a median time to first decision of 18 days and a median time to publication of 39 days. The editors would like to express their sincere gratitude to the following reviewers for their cooperation and dedication in 2018:
Abdeen, AyeshaLindquist, LeeAgogo, George O.Lodovici, MauraAllen, Jacqueline E.Londos, Elisabet Y.Amini, RezaLopez-Valcarcel, Beatriz GonzalezAntin, Jonathan FrankLorig, KateArcher, NormLundh Hagelin, CarinaArmstrong, Nicole M.Macijauskienė, JūratėAschenbrenner, AndrewManzo, CiroAustrom, Mary GuerrieroMarcus, Esther-LeeBailey, CatherineMarcusson, JanBalogun, RasheedMarkle-Reid, MaureenBanerjee, SudipMarottoli, Richard A.Beer, Jürg H.Master Sankar Raj, VimalBern-Klug, MercedesMathieson, StephanieBernstein, MelissaMcKinstry, BrianBirmingham, WendyMegaloikonomos, Panayiotis D.Biswas, DwaipayanMehlsen, Mimi YungBliemel, ChristopherMelgaard, DorteBordonaro, MichaelMiglinas, MariusBossen, Ann L.Miles, AnnaBoufous, SoufianeMilla, SaajanahoBraam, ArjanMiotla, PawelBrouwer, Wiebo H.Mistiaen, WilhelmBrzoska, PatrickMixon, Amanda S.Bujnowska-Fedak, Maria MagdalenaMonekosso, DorothyBullo, ValentinaMontero-Odasso, ManuelCancela Carral, José M^ᵃ^Mooijaart, Simon P.Capecci, ElisaMorgan, DeidreCarlsson, MartinMorino, KatsutaroCasas, OscarMulheren, RachelCassarino, MaricaMurata, ChiyoeCatania, GianlucaMusselwhite, CharlesCerejeira, JoaquimNamasivayam-MacDonald, AshwiniChan, Keith T.Neumann, ThomasChan, DanielNg, KwokChandler, Melanie J.Norman, Gregory J.Chapman-Novakofski, KarenO’Donovan, AnitaChen, Mei-LanOstaszkiewicz, JoanChruściel, PawełOzawa, MioClavé, PerePachana, Nancy A.Coenen, MichaelaPaire-Ficout, LaurenceCollard, Rose M.Papacleovoulou, GeorgiaCook, PaulPapazafiropoulou, AthanasiaCorcos, Daniel M.Patel, Harnish P.Corica, FrancescoPavlou, DimosthenisCosentino, Stephanie A.Pedersen, Mette MereteDa Silva, Rubens A.Pekmezovic, TatjanaDamsgaard, Else MariePereira, AnaDavis, JenniferPerna, SimoneDe Koning, Elisa J.Petretto, DonatellaDi Bari, MauroPisegna, Jessica M.Dibaba, DanielPotter, KathleenDickerson, AnnePoulsen, IngridDietsch, Angela M.Pownall, SueDjärv, TheresePravst, IgorDonato, RosarioPulle, Ranjeev ChrysanthDreher, MarkPutnam, MichelleDroz, Jean-PierreRay, Robin A.Dunning, TrishaRegan, JulieEdes, Thomas E.Ribeiro, RosileneEdmonds, EmilyRobinson, LouiseEllis, Robert J.Rojo Del Olmo, LuisEsposito, CiroRosenzweig, AllisonEwen, Heidi H.Rowe, MeredethEysel-Gosepath, KatrinSadowski, Cheryl A.Facal, DavidSalkini, MohamadFalkenstein, MichaelSalvi, FabioFalvey, JasonSantana-Mancilla, Pedro C.Farina, NicolasSantulli, GaetanoFisk, Malcolm J.Sanz-Paris, AlejandroFranzen, Aaron B.Saotome, MasaoFurman, Christian D.Saraux, AlainGaffo, Angelo L.Schmidt, CarolineGagnon, SylvainSchofield, PatriciaGao, QiSchubert, Cathy C.Gazdag, GaborSchunk, MichaelaGentry, MelanieShimada, MasakoGeorge, StaceyShune, SamanthaGijón Nogueron, GabrielSingh, InderpalGoldzweig, GilSixsmith, JudithGomez-Perez, Sandra L.Slee, AdrianGordon, MatthewSmithard, David G.Gu, YianSnih, Soham AlGurrapu, SreeharshaStafford, GloriaHaboubi, NadimSudbury-Riley, LynnHajjar, EmilySund Levander, MärtaHale, BeverleySuurmond, JeanineHaugland, TrudeSwaine, Ian L.Hemmy, LauraSwift, Hannah J.Henderson, EmilySyrjälä, Anna-MaijaHinault, ThomasTakahashi, KoichiroHinyard, Leslie J.Tarazona-Santabalbina, Francisco JoséHirasaka, KatsuyaTaylor, LynneHo, RogerThingstad, PernilleHobbelen, HansThomas, ChristineHollaar, VanessaThomas, Elizabeth AnneHossain, Mohammad ZakirThomas-Hawkins, CharlotteHshieh, Tammy T.Tiidus, PeterHunt, Linda A.Tomita, Machiko R.Huseth-Zosel, Andrea L.Triggiani, VincenzoInvernizzi, MarcoTroutman-Jordan, MeredithJäncke, LutzTsakiris, Dimitrios A.Jankowski, Catherine M.Tunuguntla, HariKanbayashi, TakashiUnni, Elizabeth J.Kane, RosalieVan Der Putten, Gert-JanKarthaus, MelanieVan Hoof, JoostKarvonen-Gutierrez, Carrie A.Vandenberg, Ann E.Keevil, Victoria L.Vaucher, PaulKevern, PeterVerin, EricKim, Won HoVincensi, BarbaraKinosian, BruceVisser, AnitaKnoefel, FrankWark, StuartKo, DamiWashington, TiffanyKotronoulas, GrigoriosWeber, DanielaKratzke, CynthiaWest, Jessica S.KUMAR, PRAKASHWhitley, EliseLafont, SylvianeWieland, G. DarrylLageman, SarahWilliams, ElizabethLamont, Robyn M.Wong, RogerLarner, Andrew J.Xiang, XiaolingLauretani, FulvioYang, YongLawrenson, RossYannis, GeorgeLee, HyokiYoung, JanetteLeng, Xiaoyan IrisYu, Tsung-HsienLeslie, PaulaZanini, MilkoLim, Wee ShiongZhu, LinLim, KarenZuidersma, MarijLindberg, GabriellaZwingmann, Christian

